# Beyond comorbidity counts: multidimensional extensions to cardiometabolic multimorbidity and perioperative neurocognitive

**DOI:** 10.1097/JS9.0000000000002992

**Published:** 2025-07-08

**Authors:** Wenxin Gao, Dipeng Li, Mao Wu

**Affiliations:** a The First Affiliated Hospital, Zhejiang Chinese Medical University, Hangzhou, Zhejiang, People’s Republic of China; b Nanjing University of Chinese Medicine, Nanjing, Jiangsu, People’s Republic of China; c Department of Trauma and Orthopedics, Wuxi Afliated Hospital of Nanjing University of Chinese Medicine, Wuxi, Jiangsu, People’s Republic of China

*Dear Editor,*

We commend Wang et al. for their insightful prospective cohort study, “Association of cardiometabolic multimorbidity with postoperative delirium and three-year mortality in patients undergoing knee/hip arthroplasty”^[1]^. This work advances perioperative medicine by establishing cardiometabolic multimorbidity (CMM) as a novel risk factor for postoperative delirium (POD), mediated via cerebrospinal fluid (CSF) T-tau, and revealing mortality disparities among CMM subgroups. The integration of CSF biomarkers with clinical outcomes provides a mechanistic foundation for risk stratification.We have been assured that the article complies with TITAN Guidelines 2025-governing declaration and use of AI^[2]^.

We propose extensions to amplify the clinical and research impact of these findings. First, neurovascular coupling mechanisms warrant exploration. CMM-driven endothelial dysfunction may impair cerebral blood flow regulation, exacerbating tau pathology, and POD susceptibility. Recent evidence shows that vascular dysregulation accelerates blood-brain barrier breakdown, facilitating neurotoxic metabolite entry (e.g., fibrinogen) that potentiates tau phosphorylation^[3]^. Multimodal prehabilitation targeting endothelial health (e.g., omega-3 supplementation, aerobic conditioning) could mitigate this pathway.

Second, digital phenotyping of CMM trajectories may enhance dynamic risk prediction. Wearable sensors capturing real-time glucose variability, heart rate turbulence, and nocturnal hypoxemia could quantify CMM severity more sensitively than diagnostic counts. Templeton et al demonstrated that digital health technologies enable more precise monitoring of neurocognitive decline and facilitate personalized treatment for patients^[4]^. Integrating such data with CSF biomarkers may refine POD risk algorithms.

Third, the pro-inflammatory nexus in diabetes-CHD comorbidity merits investigation. This subgroup’s high mortality may stem from IL-6/CRP synergy, driving neuronal insulin resistance and tau hyperphosphorylation. Research demonstrates that inhibition of inflammatory cytokines, particularly IL-6, significantly reduces oxidative stress-induced brain damage in diabetic mice, ameliorating associated cognitive impairment^[5]^. Translating these to perioperative settings – particularly for diabetes-CHD patients – could test causality in the CMM-POD-mortality axis.

Lastly, gut–brain axis modulation represents an unexplored opportunity. CMM correlates with dysbiosis-induced endotoxemia, elevating systemic LPS and promoting microglial activation. In Alzheimer’s disease rat models, fecal microbiota transplantation attenuates tau pathology through modulation of gut microbial composition^[6]^. Preoperative probiotics or dietary fiber interventions could be tested in high-CMM cohorts.

Wang et al’s mediation model (T-tau explaining 11% of CMM-POD risk) implies additional pathways. We recommend exploring:

Mitochondrial dysfunction: CMM impairs hippocampal bioenergetics;

Surgical stress granularity: Intraoperative hemodynamic fluctuations may disproportionately affect CMM patients. Beat-to-beat blood pressure analytics could identify vulnerability thresholds.

These strategies may transform CMM from a risk marker to a modifiable target, advancing precision perioperative care.

## Data Availability

Not applicable.
